# Supplementary material for: Effect of intraoperative systemic magnesium sulphate on postoperative Richmond Agitation-Sedation Scale score after endovascular repair of aortic aneurysm under general anesthesia: A double-blind, randomized, controlled trial
Source: PLoS One. 2023 Feb 7;18(2):e0281457. doi: 10.1371/journal.pone.0281457 (PMC9904453; doi:10.1371/journal.pone.0281457)
Supplement: S1 File — (DOCX) [file pone.0281457.s001.docx]

Supplement 1 for the study, Effect of intraoperative systemic magnesium sulphate on postoperative Richmond Agitation-Sedation Scale score after endovascular repair of aortic aneurysm: a double-blind, randomized, controlled trial.

The reasons for the difference of the dates of the registrations between our IRB and jRCT on the WEB.

We are really sorry for the difference of the registration for our study between our IRB and jRCT on the WEB. We are really sorry for this failure of the registration procedure. We misunderstood that we had finished all registration procedures with the meeting with jRCT staffs and we got the approved paper on 18 October 2018 from our university IRB. However, actually, we should have sent the documents that we should have printed out from WEB site, to the jRCT and after getting the letter from us, the procedure would have done and jRCT would have registered our study on the WEB. We thought that our protocol had already been approved, therefore, we started patient recruitment. We did not notice that on jRCT WEB our protocol had not approved yet. As soon as possible after noticing it, we tried to confirm the popper procedure and send the needed documents to jRCT. As the results, the difference between the text and URL on the registration dates happened. However, the authors confirm that all ongoing and related trials for this drug and intervention are registered.
