# Supplementary material for: Effect of intraoperative systemic magnesium sulphate on postoperative Richmond Agitation-Sedation Scale score after endovascular repair of aortic aneurysm under general anesthesia: A double-blind, randomized, controlled trial
Source: PLoS One. 2023 Feb 7;18(2):e0281457. doi: 10.1371/journal.pone.0281457 (PMC9904453; doi:10.1371/journal.pone.0281457)
Supplement: S2 File — (DOCX) [file pone.0281457.s002.docx]

臨床研究計画書

研究課題名：

大血管ステント留置術後鎮静度RASSスコアに対するマグネシウム投与の有用性に関する単施設二重盲検ランダム化コントロール試験

診療科：麻酔科学講座

診療科長：教授　藤原　祥裕

研究責任医師：教授（特任）　藤田　義人

作成年月日：2018 年7月4日

**1.研究担当者**

責任者：愛知医科大学医学部麻酔科学講座 教授　　　　　藤田義人

分担者：愛知医科大学医学部麻酔科学講座 教授　　　　　藤原祥裕

分担者：愛知医科大学医学部麻酔科学講座 専修医　　　　金森春奈

分担者：愛知医科大学医学部麻酔科学講座 専修医　　　　石原亮太

**2.臨床研究の実施体制**

本研究は研究機関の長である愛知医科大学病院病院長の許可を受けて実施されている。研究機関は、愛知医科大学麻酔科学教室で研究担当者は上記である。

**3. 研究の背景**

　近年、ICUにおいてせん妄の発症は患者死亡率増加の独立した因子とて認識されているが、その予防、治療で有用な薬剤、および方法として認められたものはない。

　一方で、手術中からのマグネシウム(硫酸Mg補正液1mEq/ml)投与は、合併症を起こすことなく安全に、術中の痛みの軽減（Br J Anaesth 2008; 100: 397-403）、術後の鎮痛薬の軽減（Br J Anaesth 2006; 96: 444-9-403）、術後の撹乱（Eur J Anaesthesiol 2017; 34:658–664）などに有効であることが先行の研究で報告されている。しかし、術後せん妄に対する効果は明らかではない。愛知医科大学麻酔科では、以前別の観察研究で、大血管ステント術後にせん妄の発生が多く、50％近くにせん妄が認められることを把握している。

　そこで、同手術術後において、適度の鎮静レベルを得る効果とせん妄予防の効果の可能性のある術中マグネシウム投与の有用性を明らかにしたい。マグネシウムと比較する薬剤は、せん妄、鎮静度に有用であると確立されたものはないため、生理食塩水を用いたプラセボを比較対象群とする。この研究を今後の術後患者管理の鎮静の質の向上に役立てたい。

当該臨床研究に用いる医薬品に関する情報：

1. 名称：硫酸Mg補正液1mEq/ml
2. 投与経路、用法、用量および投与期間：硫酸マグネシウム30mg/kg/hで1時間、その後麻酔終了まで（麻酔時間が4時間を超える場合は4時間まで）、10mg/kg/hで投与する
3. 対象集団：60才以上で愛知医科大学病院で大血管（胸部及び腹部大動脈瘤に対してステント留置手術を受ける患者
4. 当該医薬品の有効性安全性：手術中からのマグネシウム(硫酸Mg補正液1mEq/ml)投与は、合併症を起こすことなく安全に、術中の痛みの軽減、術後の鎮痛薬の軽減、術後の撹乱などに有効であることが先行の研究で報告されている（Eur J Anaesthesiol 2017; 34:658–664など）。

**4.研究の目的**

　今回、手術中からマグネシウム投与がせん妄の発生に影響を与えるかを、preliminaryな研究として、プライマリーのアウトカムとして、せん妄評価の際に使用されるRASS(Richmond agitation-sedation score)を使用する（Lancet 2016; 388: 1893–902）。RASSスコアは、-5（昏睡状態）から0（覚醒し落ち着いている）さらに+4（好戦的）までの10段階で評価する。その+の項目は「過活動型せん妄の興奮の程度」を評価する役割を果たす。そのスコアが統計学的に有意差を持って下げることができるかどうかを検討する。二次のアウトカムとして、せん妄の発生率（CAM-ICU）、鎮痛スコア(NRS、BPS)、鎮痛薬使用、GICU入室期間などを検討する。術中からのマグネシウム投与が、術後のより適切な鎮静度をもたらすかどうかを検討する。

**5.研究の内容**

　方法：対象術前患者に対し『研究の目的、研究の方法、研究への参加予定人数・期間、予測される臨床上の危険性及び不利益、研究への参加は自由で，参加しなくても不利益を一切被らないこと、研究の参加に同意した場合でも，いつでも撤回できること、プライバシーは一切公開されず，守られること、および研究責任者の所属・職名・氏名・連絡先』を説明し、当該研究に対するインフォームド・コンセントをえる。

　手術前検査および診察情報により、精神疾患、心疾患、腎機能障害、糖尿病、高血圧、高コレステロール血症、高尿酸血症、喫煙など既往歴の有無を対象症例で確認し、その詳細を記録する。この時点からデータは匿名化を行い、本研究を開始する。本研究では、作成された麻酔記録、術前の検査データや投薬内容などは研究開始直後から匿名化する。カルテや麻酔記録から抽出した臨床データを保管する場合も匿名化したままとし、臨床および解析データは愛知医科大学麻酔科学講座医局に保管する。データは研究期間終了後も愛知医科大学麻酔科医局に保管するが、データを別の目的に使用する際には本学倫理委員会に改めて申請し承認を受け、また、被検者の同意を得た上で使用する。

　本研究に参加する同意を得た患者をコンピュータで作成した乱数表に基づき（A）マグネシウム群と（B）コントロール群に振り分ける。乱数表はエクセルを用いてあらかじめ作成し、同意が得られた患者を順番に各群に振り分ける。（A）群では手術室入室後、静脈ルート確保後、薬剤部にあらかじめダブルブラインドになるようにシリンジに充填していただいた硫酸マグネシウム入りの薬剤の投与を開始する。薬剤は先行の研究で安全性と有用性が明らかになっている、硫酸マグネシウム30mg/kg/hで1時間、その後麻酔終了まで（麻酔時間が4時間を超える場合は4時間まで）、10mg/kg/hで投与する。通常の麻酔薬、プロポフォール及び、レミフェンタニル、ロクロニウムで全身麻酔を導入する。術中の麻酔維持は、酸素、空気、デスフルラン、フェンタニル、レミフェンタニルによる全身麻酔で維持する。（B）群では、プラセボとして、生理食塩水を充填したシリンジにて同様のスピードで麻酔終了まで投与する。術中の全身麻酔管理は（A）群と同様に行う。手術術者、麻酔管理者、手術室スタッフ、GICU看護師はどちらの群に属していうるかは把握できない。また、手術終了後に覚醒不良だけでなくいかなる理由でも抜管ができなかった場合は、調査の対象から除外する。

　主要評価項目：総合集中治療室（GICU）入室時の鎮静スコア（RASSスコア）

　副次評価項目：せん妄の発生率（CAM-ICU）、鎮痛スコア(NRS、BPS)、GICU入室後1時間、6時間、12時間、24時間、GICU入室後24時間までの鎮痛薬の使用、GICU入室期間

　術後の評価項目は、評価項目用紙をすべての症例にあらかじめ用意し記入をGICU看護師にお願いする。2群間で(1)GICU入室後のRASSスコアと鎮痛スコア、(2)GICU入室後1時間、6時間、12時間、24時間後のRASSスコア及び疼痛スコア、(3)GICU入室後24時間までの鎮痛薬使用、(4)GICU入室期間をそれぞれ比較する。その他、術後採血データ、術後24時間の心拍数、血圧、酸素飽和度を参照する。

本研究は二重盲検の前向き試験である。

研究の手順は以下のようである。

手術後

手術中

手術前

6. 臨床研究の対象者の選択及び除外並びに臨床研究の中止に関する基準

　被験者：愛知医科大学病院で大血管（胸部及び腹部大動脈瘤に対してステント留置手術を受ける患者約60 人。

　選択基準：60才以上で大血管（胸部及び腹部大動脈瘤に対してステント留置手術を受ける患者。

　除外基準：緊急手術患者。重篤な心機能障害を持つ患者。クレアチンが2を超える、または透析を行っているような重篤な腎機能障害を持つ患者。重篤な肝機能障害を持つ患者。重篤な呼吸機能障害をもつ患者。重度の双極性障害、自殺念慮、自殺企図など重度の精神病状態にある患者またはその既往のある患者、あるいは認知症と診断された患者。研究者が調査対象として不適切と判断した患者。また、術後に覚醒不良だけでなくいかなる理由でも抜管ができなかった場合は、調査の対象から除外する。

　中止基準：血圧、心拍数などのバイタルサインの異常により麻酔管理の維持が難しく、その原因としてマグネシウム投与が考えられる場合。

7. 臨床研究の対象者に関する治療

　疾病等が発生した場合の対応に関する手順書を別に示す。研究により被験者に健康被害等の有害事象が発生した場合、保険診療により対応する。これまで別のoutcomeである鎮痛の目的で硫酸マグネシウムを投与した臨床研究が行われてきた。今回の研究では、これらの先行研究で使用された投与量を基準にほぼ同量の投与量としているが、先行研究では合併症の報告はなかった。

8. 有用性の評価

　主要評価項目：総合集中治療室（GICU）入室時の鎮静スコア（RASSスコア）

　副次評価項目：せん妄の発生率（CAM-ICU）、鎮痛スコア(NRS、BPS)、GICU入室後1時間、6時間、12時間、24時間、GICU入室後24時間までの鎮痛薬の使用、GICU入室期間

　術後の評価項目は、評価項目用紙をすべての症例にあらかじめ用意し記入をGICU看護師にお願いする。2群間で(1)GICU入室後のRASSスコアと鎮痛スコア、(2)GICU入室後1時間、6時間、12時間、24時間後のRASSスコア及び疼痛スコア、(3)GICU入室後24時間までの鎮痛薬使用、(4)GICU入室期間をそれぞれ比較する。その他、術後採血データ、術後24時間の心拍数、血圧、酸素飽和度を参照する。

　得られたデータはデータ解析ソフトを使用し、primary outcomeは、等分散性を確認後、ｔ検定、もしくはマンホイットニーU検定により群間差を比較する。また、secondly outcomeもそれぞれの評価項目にふさわしい統計法を用いて評価する。

9. 安全性の評価

　麻酔投与中に関しては、血圧、心拍数などのバイタルサインの異常により評価する。麻酔管理の維持が難しく、その原因としてマグネシウム投与が考えられる場合は中止する。術後は、高マグネシウム血症の合併症である、熱感，血圧降下，中枢神経抑制，呼吸麻痺などのマグネシウム中毒症状が術後1日以内に認められた場合、もしくは特定臨床研究の実施に起因すると疑われる疾病、障害、若しくは死亡、又は感染症に加え検査値の異常が術後1週間に以内に認められた場合、以後の研究は中止とする。

10. 統計的な解析

　先行研究で扁桃摘出術後のagitationの発生をマグネシウム投与により減少させることができたという論文では、RASSスコアは、群間での差は1-2であった（*Eur J Anaesthesiol 2017; 34: 658-664*）、他の術後せん妄に関する論文でも群間0.5-1である。又、RASSの標準偏差は0.3-0.6であった。そこで群間のRASSスコアの差を、0.3-1とし、標準偏差を0.3-0.6、αエラーを0.0５、powerを0.8としてサンプルサイズを計算すると、必要症例数は、多く見積もって23と計算される。そのため片群30とした。

　片群15程度行った時に中間解析をおこない再度必要症例数を計算する。もし中間解析の時点で有意差が出た場合は、オブライエン-フレミング法の有意水準(0.005)を持って有意と判断する。逆に逆効果（マグネシウム群の方が鎮静度が低い結果、安全性に問題あり）は、0.05の有意水準を持って有意と判断する。また、非有効性（無益性）については、再度計算した必要症例数が当初予定の倍の症例数が必要となった場合とし、研究を断念する。

11. 原資料等の閲覧

　研究責任医師が、臨床研究に関連するモニタリング、監査並びに認定臨床研究審査委員会及び規制当局の調査の際に、原資料等の全ての臨床研究関連記録を直接閲覧する。

12. 品質管理および品質保証

　モニタリングは別にモニタリングに関する手順書を示す。

13. 倫理的な配慮

　当該研究において研究の対象者には、特に利益、不利益となることはない。

14. 記録の取り扱い保存

　記録の保管期間は、研究終了の報告後５年，又は結果の最終の公表について報告された日から３年を経過した日のいずれか遅い日までとする。保管場所は、施錠可能である愛知医科大学麻酔科医局内とし、破棄は匿名化した状態でシュレッダーにて裁断する。

14. 臨床研究の実施に係る金銭の支払及び補償

臨床研究の実施に際して金銭の支払いはない。補償に関して研究用に保険に加入することはない。補償は医療保険内とする。

15. 臨床研究に関する情報の公表

　厚生労働省が整備するデータベース（以下「jRCT」（Japan Registry of Clinical Trials）という。）に記録し、公表する。

16. 臨床研究の実施期間

　倫理審査承認日より2020年12月31日までを予定する。

17. 臨床研究の対象者に対する説明及びその同意

　説明書及び同意書を別紙に示す。

18. 前各号に掲げるもののほか、臨床研究の適正な実施のために必要な事項

利益相反管理計画を別に作成する。

19.予想される医学上の貢献

　手術中からマグネシウム投与がせん妄の発生に影響を与えるかを、プライマリーのアウトカムとして、鎮静の評価として確立されたRASS(Richmond agitation-sedation score)を用いて、そのスコアを下げることができるかどうかを検討されることにより、現在までせん妄対しての治療および予防の方法がない中、投与による合併症がないマグネシウム投与がせん妄予防への可能性があるかどうかが明らかにされる。

20.当該臨床研究に参加することにより期待される利益及び起こり得る危険並びに必然的に伴う心身に対する不快な状態について

　本研究に先立って同意を得る際には，何ら不利益をうけることなく自由意思で，研究への参加・不参加を選択できる。また，その研究参加の意思表示を撤回できることを保障し、新たな研究のために既存の資料を利用する場合、資料提供者の同意を得ることが困難な場合，情報公開により被験者となることを拒否できる機会を設けることを約束する。本研究では、被験者のプライバシー及び個人情報の保護に努める。

21.当該臨床研究に係る資金源、起こり得る利害の衝突及び研究者等の関連組織との関わり

　本研究は、講座研究費を資金源にする予定であるが、将来獲得した科学研究費が使用される可能性は否定しない。なお、本研究に企業等は関与しない。

　利益相反管理計画に基づき本研究で使用する硫酸マグネシウムを製造・販売している大塚製薬株式会社との間に研究資金の提供や研究者個人への謝金の支払いなど利益相反は存在しない。

22.本研究の問い合わせ先

愛知医科大学医学部麻酔科学講座　教授（特任）　藤田　義人

0561-62-3311（内線77837）

E-mail：fujita.yoshihito.823@mail.aichi-med-u.ac.jp
