# Supplementary material for: Effect of intraoperative systemic magnesium sulphate on postoperative Richmond Agitation-Sedation Scale score after endovascular repair of aortic aneurysm under general anesthesia: A double-blind, randomized, controlled trial
Source: PLoS One. 2023 Feb 7;18(2):e0281457. doi: 10.1371/journal.pone.0281457 (PMC9904453; doi:10.1371/journal.pone.0281457)
Supplement: S3 File — (DOCX) [file pone.0281457.s003.docx]

The translation of the main points of the protocol of Effect of intraoperative systemic magnesium sulphate on postoperative Richmond Agitation-Sedation Scale score after endovascular repair of aortic aneurysm: a double-blind, randomized, controlled trial

The protocol

Title: Effect of intraoperative systemic magnesium sulphate on postoperative Richmond Agitation-Sedation Scale score after endovascular repair of aortic aneurysm: a double-blind, randomized, controlled trial

Main conducting doctor: Yoshihito Fujita

Date: 4^th^ July, 2018

1. Background

Delirium is common in vascular surgery settings, and associated anesthesia management and intra-operative drugs have attracted substantial interest. Intraoperative magnesium has the effect of reducing intra-operative and postoperative opiate requirement and pain. Recently it was reported to decrease postoperative agitation, which might suggest that magnesium could have an effect on not only pain relief but also sedative level. However, its effect on postoperative sedation and delirium is unclear. We investigated its effect on the postoperative Richmond Agitation-Sedation Scale (RASS) score, pain score (numerical rating scale: NRS), delirium (Confusion Assessment Method for the Intensive Care Unit: CAM-ICU), and length of ICU stay, and hypothesized that magnesium infusion would reduce RASS score after surgery for endovascular aortic aneurysm repair (EVAR).

Information using drugs:

I; magnesium sulphate; Otsuka Pharmaceutical, Japan) 1mE/ml

II; infusion method:

The pharmacist prepared the magnesium-solution syringes by mixing magnesium sulphate with 0.9% saline according to the actual body weight or ideal body weight. To avoid overdose of magnesium administration to obese patients, we applied ideal body weight (body mass index: 22) when the actual body weight was greater than the ideal one. We used magnesium sulphate solution containing 123 mg of magnesium per 1 ml. For each patient, a total dose of magnesium sulphate of 60 mg•kg^−1^ was diluted to 60 ml. Patients in the magnesium group received an initial intravenous loading dose of 30 mg•kg^−1^ over 1 h, followed by a continuous infusion of 10 mg•kg^−1^ h^−1^ for the duration of surgery. The infusion was terminated when the duration exceeded 4 h in total, and was delivered using a syringe pump. The control group received an equivalent volume of 0.9% saline. These syringes were delivered to the attending anesthesiologist by another pharmacist.

III; participants:

Patients of either sex aged 60 years or older who were scheduled for endovascular repair of abdominal or thoracic aortic aneurysm under general anesthesia at our hospital were eligible.

IV; Effectiveness and safety of the using drugs:

We used the previous protocol for obtaining a positive effect on agitation, magnesium sulphate 30 mg bolus followed by 10 mg•kg^−1^ intraoperative infusion, targeting a total 60 mg•kg^−1^, which demonstrated in the FESS study that magnesium administration could reduce the incidence of agitation and there were no side effects for the participants.

1. Purpose

We investigated its effect on the postoperative Richmond Agitation-Sedation Scale (RASS) score, pain score (numerical rating scale: NRS), delirium (Confusion Assessment Method for the Intensive Care Unit: CAM-ICU), and length of ICU stay, and hypothesized that magnesium infusion would reduce RASS score after surgery for endovascular aortic aneurysm repair (EVAR). The primary outcome was difference of Richmond Agitation-sedation Scale; RASS score between two groups: we examine whether infusion of magnesium reduces RASS score. The secondary outcomes were Incidence of delirium, analgesia score (numerical rating scale; NRS, behavioral pain score; BPS), use of analgesic drugs, General ICU stay.

1. Materials and methods

Written informed consent was obtained from all participants. Patients of either sex aged 60 years or older who were scheduled for endovascular repair of abdominal or thoracic aortic aneurysm under general anesthesia at our hospital were eligible. Exclusion criteria were: (1) patients who required emergency surgery; (2) patients with severe complications, including cardiac, renal (including dialysis), blood, lung, liver, or life-threatening disease; (3) patients with serious complications arising from psychological illness (including bipolar disorder, suicide intent). Severe renal dysfunction was defined as estimated glomerular filtration rate (eGFR) less than 30 ml•min^−1^•1.7 m^−2^ or utilization of hemodialysis.

Patients were assigned randomly to one of two groups to receive intra-operative magnesium (magnesium sulphate; Otsuka Pharmaceutical, Japan) (magnesium group) or 0.9% saline solution (control group). A computer-generated randomization program was used. The independent anesthesiologist who was not involved in anesthesia management and outcome evaluation provided a pharmacist with sealed envelopes which included patient identification, group allocation and body weight, and the attending pharmacist prepared the colorless coded solutions in transparent syringes. The codes were kept confidential by the independent anesthesiologist until completion of the study. The pharmacist prepared the magnesium-solution syringes by mixing magnesium sulphate with 0.9% saline according to the actual body weight or ideal body weight. To avoid overdose of magnesium administration to obese patients, we applied ideal body weight (body mass index: 22) when the actual body weight was greater than the ideal one. We used magnesium sulphate solution containing 123 mg of magnesium per 1 ml. For each patient, a total dose of magnesium sulphate of 60 mg•kg^−1^ was diluted to 60 ml. Patients in the magnesium group received an initial intravenous loading dose of 30 mg•kg^−1^ over 1 h, followed by a continuous infusion of 10 mg•kg^−1^ h^−1^ for the duration of surgery. The infusion was terminated when the duration exceeded 4 h in total, and was delivered using a syringe pump. The control group received an equivalent volume of 0.9% saline. These syringes were delivered to the attending anesthesiologist by another pharmacist.

No patient received premedication. On arrival at the operating room, standard monitoring of pulse oximetry (SpO^2^), noninvasive blood pressure (NIBP), ECG, and heart rate (HR) was implemented. A 22- or 20-gauge cannula was inserted into a peripheral vein. A 22-gauge catheter was inserted into the left radial artery and connected to a transducer for direct measurement of arterial pressure throughout the procedure. Inspired and end-tidal concentrations of oxygen and desflurane, and end-tidal CO_2_ concentration were monitored intra-operatively. An acceleromyograph (TOF-Watch SX; Organon, Ireland) was attached to stimulate either ulnar or facial nerve to measure the response of adductor pollicis or corrugator supercilii, respectively, to monitor neuromuscular block, because the administration of magnesium can extend the effect of non-depolarizing neuromuscular relaxants. The Bispectral Index (BIS) was also monitored by an Aspect XP A2000 device (Aspect Medical Systems, Minneapolis, MN, USA) to prevent awareness, a possible contributing factor to postoperative agitation.

Anesthesia was induced with propofol 0.5 to 2 mg•kg^−1^, remifentanil 0 to 0.3 µg•kg^−1^•min^−1^, and fentanyl 0 to 200 mg, followed by rocuronium 0.6 to 0.9 mg•kg^−1^ to facilitate tracheal intubation. Mechanical ventilation was then started, and anesthesia was maintained with an inspired desflurane in air/O_2_ mixture. During the operation, rocuronium 10 mg was administrated when the patient showed two twitch responses of train-of-four (TOF) stimuli. The anesthesiologist adjusted the concentration of desflurane to maintain the BIS value between 40 and 60. Mean arterial blood pressure was monitored continuously and maintained within 70% and 130% of the value before induction of anesthesia. Bolus injection of ephedrine or phenylephrine was used to treat hypotension, which was less than 90 mmHg of systolic blood pressure during anesthesia. Bolus injection of nicardipine hydrochloride was used to treat hypertension, which was more than 140 mmHg of systolic blood pressure during anesthesia. In this study we did not provide a strategy of permissive hypotension. If the attending anesthesiologist needed drugs for postoperative analgesia, acetaminophen 1000 mg and/or flurbiprofen axetil 50 mg was given intravenously during the operation.

All the EVAR surgeries were performed with radiological imaging in a hybrid operating room attended by not only vascular surgeons but also radiologists who were specialists in this field, thus ensuring that all patients obtained sufficient cerebral blood perfusion during surgery. For anticoagulation, routinely 3000 units of intravenous unfractionated heparin were administered during surgery, and the administered heparin was titrated to maintain 150 to 200 seconds of activated coagulation time. In our EVAR surgeries there was no use of additional oral anticoagulation drugs before and after surgery. However, if the patients used oral anticoagulation drugs before surgery for any reason, the drugs were stopped before surgery and restarted 24 h after surgery. As for a radiological contrast, the minimum volume of a radiological contrast was administered to perform surgical procedure by vascular surgeons and radiologists. According to our antibiotics protocol for EVAR surgery, 1g of cefazolin was administered before starting surgery, and after surgery 1 g of the same antibiotic was given twice a day for 48 h postoperatively.

The infusion of magnesium or saline was terminated when the operator started to close the wound. We set the upper limit of the total infusion period to 4 h for 60 mg•kg^−1^. At the end of surgery, sugammadex was given intravenously to reverse residual neuromuscular block. The dose of sugammadex was at least 2 mg•kg^−1^ for moderate block (when the second twitch in response to TOF stimuli was observed) and 4 mg•kg^−1^ for deep block (when the twitch response reached a post-tetanic count of at least 1). When the attending anesthesiologist assessed that the patient had recovered sufficiently from anesthesia, the tracheal tube was removed. Patients were then transferred to the postoperative ICU. An oxygen face mask delivered a flow rate of 3 l•min^−1^.

We recorded total doses of fentanyl, remifentanil and magnesium sulphate, duration of anesthesia, duration of operation, amount of bleeding, amount of crystalloid fluid infusion (contains Mg 1 mmol•l^−1^), total plasma magnesium value, and ionized plasma magnesium value, using an ion-selective analyzer (Stat Profile Prime ES Comp Analyzer; NOVA Biomedical, Waltham, MA, USA) before induction, after transfer to the postoperative ICU, and on the day after surgery at 6 a.m.

In the ICU, SpO_2_, HR and arterial BP were monitored, and levels of agitation and pain were assessed and recorded. Discharge criteria from the ICU were stable vital signs, free of major complications that require intensive care. Delirium was defined as CAM-ICU positive. RASS score (0, alert and calm; +1, restless; +2, agitated; +3, very agitated; +4, combative; −1, drowsy; −2, light sedation; −3, moderate sedation; −4, deep sedation; −5, unrousable1) was assessed in the ICU by a blinded observer, an attending ICU nurse. Pain scores were assessed using an NRS pain score as rated by the patient, where 0 represents no pain and 10 is the worst imaginable pain. Blinded nurses in the ICU assessed and recorded CAM-ICU, RASS and NRS at the postoperative ICU transfer (time 0) and 1 h, 6 h and 24 h later. If the patient was discharged from the ICU within 24 h, the nurse recorded those values of sedative level at the time of ICU discharge. Use of acetaminophen and dexmedetomidine, and the length of stay in ICU, were also recorded.

Primary outcome of this study was postoperative RASS score at postoperative ICU transfer. Secondary outcomes were incidence of delirium defined using CAM-ICU, pain according to NRS, use of analgesic drugs, and length of ICU stay. Therefore, attending surgeons, attending anesthesiologists, attending operative staff, attending ICU staff, and outcome evaluators could never know which group to which the patient belonged.

1. Evaluations for the effectiveness and statistical analysis.

We calculated the sample size for the primary analysis on the basis of differences seen in the previous study. In the study about reducing postoperative agitation in patients undergoing functional endoscopic sinus surgery (FESS), the difference in RASS scores between two groups was 1 to 2 during the observation period.^9^ In this previous study, the SD was not presented. In another retrospective observational study (not published), we investigated the rate of hyperactive delirium among patients who underwent EVAR surgery under general anesthesia in our hospital in the same setting. We reported RASS scores of 0.25±0.5 and 58 cases of hyperactive delirium among 237 patients. Therefore, we estimated that score levels differed by 1 to 2 with SD of 0.5 to 1. Assuming an α level of 0.05 and 90% power, the required number of patients for each group to observe a RASS difference is 22 at most. We considered that a total workable sample size would be 30 patients to detect a difference of 1 to 2, assuming a two-tailed type I error of 5% and type II error of 10%. To allow for a 5% dropout rate, we randomly assigned 32 patients.

Data are expressed as the mean±SD or median [IQR] for non-normally distributed variables (Kolmogorov–Smirnov test), or number and percentage as appropriate. All *P* values are two-tailed. *P* values of less than 0.05 were considered significant. Quantitative variables were compared using Student’s *t* test or the Mann–Whitney *U* test where appropriate. Categorical variables are described using number (%) and were compared using χ2 test or Fisher’s exact test. Interim analysis was scheduled to take place after recording results from half of the sample size, at which point the decision to continue or stop the study would be made on the basis of valid reasons. For the calculated sample size to be deemed insufficient such reasons would include the potential magnitude of bias or the potential impact on interpretation of the results.
