# Supplementary material for: Effect of intraoperative systemic magnesium sulphate on postoperative Richmond Agitation-Sedation Scale score after endovascular repair of aortic aneurysm under general anesthesia: A double-blind, randomized, controlled trial
Source: PLoS One. 2023 Feb 7;18(2):e0281457. doi: 10.1371/journal.pone.0281457 (PMC9904453; doi:10.1371/journal.pone.0281457)
Supplement: Attachment — Submitted filename: Response to Reviewers R2_PlosOne_reply_2 20221206.docx [file pone.0281457.s007.docx]

Professor Raphael Cinotti
Academic Editor
PLOS ONE

December 21, 2022

Dear Professor Raphael Cinotti,

Re: Manuscript reference No. PONE-D-22-03920R1

Please find attached a revised version of our manuscript “Effect of intraoperative systemic magnesium sulphate on postoperative Richmond Agitation-Sedation Scale score after endovascular repair of aortic aneurysm: a double-blind, randomized, controlled trial”, which we would like to resubmit for publication as a clinical trial to the Academic Editor in PLOS ONE.

The comments of the reviewers were highly insightful. In the following pages are our point-by-point responses to each of the comments of the reviewers.

Revisions in the text are shown redlined. We hope that the revisions in the manuscript and our accompanying responses will be sufficient to make our manuscript suitable for publication in PLOS ONE.

We shall look forward to hearing from you at your earliest convenience.

Yours sincerely,

Yoshihito Fujita, MD, PhD

Address: 1-1 Karimata Yazako Nagakute, Aichi, Japan

Ph: +81-561-62-3311

Fax: +81-561-62-4866

E-mail: fujita.yoshihito.823@mail.aichi-med-u.ac.jp

Response to comments of the academic editor:

1, -please make sure you do not detail too much your methods in the introduction

*Response: Thank you for the important point for our manuscript. We tried to rewrite the content according to your suggestions.*

*We deleted “We chose the vascular surgery setting and older patient population not to decrease incidents of delirium. We planned to perform a double-blind, randomized, controlled trial and analysis of intention to treat analysis.* *We investigated its effect on the postoperative Richmond Agitation-Sedation Scale (RASS) score, pain score (numerical rating scale: NRS), delirium (Confusion Assessment Method for the Intensive Care Unit: CAM-ICU), and length of ICU stay.”* *And the word explanations of abbreviations were added to the following the sentences.*

2. -please describe the timing of evaluation of the RASS. My feeling is that "at ICU transfer" is not detailed enough. As this is the primary outcome I believe it is important to be more specific. Did you evaluate the RASS 2 hours after the end of surgery? Is the patient immediately transferred in the ICU or after extubation in the PACU?

*Response: Thank you for the important point for our manuscript. These were not detailed enough. We tried to rewrite the content according to your suggestions.*

*Actually, all patients were extubated at the operating room and were moved to the ICU. And, as soon as the patients arrived at the ICU, the ICU nurses examined vital signs and evaluated RASS, NRS and CAM-ICU. And then, we evaluated the patients 1 hr, 6 hr and 24 hr after the admission of ICU.*

*Therefore, we added the sentence “all patients were extubated at the operating room and were moved to the ICU. And, as soon as the patients arrived at the ICU, the ICU nurses examined vital signs and evaluated RASS, NRS and CAM-ICU.*

*In addition, we use the words “ICU admission” instead of “ICU transfer”.*

3. -please be more specific in the results about the adverse events: I believe that "and so on" is too blurry.

*Response: Thank you for the important point for our manuscript. These sentences were not detailed enough. We tried to rewrite the content according to your suggestions.*

*Actually, we examined existence or non-existence of arrythmia, bradycardia, hypotension, effects of muscle paralysis, and convulsion. Therefore, we rewrote*

*“During the observational period, no adverse events caused by magnesium infusion were recorded, which were existence or non-existence of arrythmia, bradycardia, hypotension, effects of muscle paralysis, and convulsion.”*

4. -please shorten your discussion. I believe that some paragraph are too putative to be discussed (level of blood Mg to be reached) or the paragraph before the limitations has too little evidence in the literature to be discussed.

*Response: Thank you for the important point for our manuscript. We tried to shorten discussion and rewrite the content according to your suggestions.*

*We deleted many sentences and two paragraph in discussion section to be easy to understand our paper and we could reduce the word counts to 3,170 from 3,620.*

Response to comments of ‘Journal Requirements:

Please review your reference list to ensure that it is complete and correct. If you have cited papers that have been retracted, please include the rationale for doing so in the manuscript text, or remove these references and replace them with relevant current references. Any changes to the reference list should be mentioned in the rebuttal letter that accompanies your revised manuscript. If you need to cite a retracted article, indicate the article’s retracted status in the References list and also include a citation and full reference for the retraction notice.

*Response: Thank you for the important point for our manuscript. We reviewed our references. And we confirmed if our cited papers are retracted. We could not find any retracted papers in our references with PubMed.gov on the WEB.*

Response to comments of the reviewers:

1. If the authors have adequately addressed your comments raised in a previous round of review and you feel that this manuscript is now acceptable for publication, you may indicate that here to bypass the “Comments to the Author” section, enter your conflict of interest statement in the “Confidential to Editor” section, and submit your "Accept" recommendation.

Reviewer #2: All comments have been addressed

Reviewer #3: (No Response)

Reviewer #4: (No Response)

2. Is the manuscript technically sound, and do the data support the conclusions?

The manuscript must describe a technically sound piece of scientific research with data that supports the conclusions. Experiments must have been conducted rigorously, with appropriate controls, replication, and sample sizes. The conclusions must be drawn appropriately based on the data presented.

Reviewer #2: Yes

Reviewer #3: No

Reviewer #4: Yes

3. Has the statistical analysis been performed appropriately and rigorously?

Reviewer #2: Yes

Reviewer #3: No

Reviewer #4: Yes

4. Have the authors made all data underlying the findings in their manuscript fully available?

The [PLOS Data policy](http://www.plosone.org/static/policies.action#sharing) requires authors to make all data underlying the findings described in their manuscript fully available without restriction, with rare exception (please refer to the Data Availability Statement in the manuscript PDF file). The data should be provided as part of the manuscript or its supporting information, or deposited to a public repository. For example, in addition to summary statistics, the data points behind means, medians and variance measures should be available. If there are restrictions on publicly sharing data—e.g. participant privacy or use of data from a third party—those must be specified.

Reviewer #2: No

Reviewer #3: Yes

Reviewer #4: Yes

5. Is the manuscript presented in an intelligible fashion and written in standard English?

PLOS ONE does not copyedit accepted manuscripts, so the language in submitted articles must be clear, correct, and unambiguous. Any typographical or grammatical errors should be corrected at revision, so please note any specific errors here.

Reviewer #2: Yes

Reviewer #3: No

Reviewer #4: Yes

6. Review Comments to the Author

Please use the space provided to explain your answers to the questions above. You may also include additional comments for the author, including concerns about dual publication, research ethics, or publication ethics. (Please upload your review as an attachment if it exceeds 20,000 characters)

Reviewer #2: (No Response)

Reviewer #3: The study size was very small. There was not enough power to determine the effect of magnesium and postoperative delirium and agitation. It really is difficult to make any useful conclusion from this study.

*Response: Thank you for the important point for our manuscript. And we could not show that Mg was effective for postoperative delirium and agitation. However, we planned a prospective RCT with calculating proper sample size. And we reached the conclusion that we could not obtain good results with the method of our protocol in this paper and we think that that is informative results in the field of Mg research.*

Unfortunately, at the same time, the goal magnesium of 60 mg/kg was not reached. From the data, this goal looks like it was never likely to be reached based on anesthesia time and the study protocol of 30 mg/kg for the first hour and then 10 mg/kg for each hour after. The maximum anesthesia time was only ~200 minutes and some cases were as short as 170 minutes.

*Response: Thank you for the important point for our manuscript. And we could not control the length of the operation. Therefore, we could not show avoid the cases with small amount of Mg. However, the same things might happen in clinical settings and measured ionized Mg had statistical differences between control group and Mg group. Next time, we have to consider that the pointed-out things would happen.*

The anesthetic protocol seems quite varied. Induction of doses of propofol and remifentanil were several fold different. There was no mention of their anesthetic targets. And postoperative use of fentanyl and dexmedetomidine were not described in the methods. While the amount of fentanyl and incidence of dexmedetomidine use look similar between groups, it is unclear if this could have affected the number of cases of delirium and agitation.

*Response: Thank you for the important point for our manuscript. In clinical setting, we thought that our results with amounts of anesthetic drugs were usual in normal anesthetic managements. And postoperative uses of fentanyl and dexmedetomidine were shown in table 4. We concluded that with these data, we reached our conclusion.*

The authors state BP goals were to maintain BP from 70% and 130%, but then nicardipine was used to treat hypertension, which was more than 140 mmHg of systolic blood pressure. It would seem for many patients 140 mmHg is not close to 130% of normal BP. This is confusing.

*Response: Thank you for the important point for our manuscript. We used nicardipine for 140mmHg of systolic blood pressure if 140 mmHg was within 130% of its normal BP for the patient.*

There is something wrong with this sentence: U10, linese of acetaminophen and dexmedetomidine,

*Response: We are really sorry for misspelling. We have rewritten this sentence.*

Page 13, paragraph 2: looks like magnesium levels are reported first for the magnesium group and then the control group except for the ionized magnesium at postoperative ICU transfer. This is confusing.

*Response: We are really sorry for misspelling. We have rewritten this sentence. We always reported the magnesium group first.*

Not sure this conclusion is determined by the study: To obtain a neurological effect, we should follow a higher-dose protocol than that employed in the present study. It is a speculation made by the authors.

*Response: Thank you for the important point for our manuscript. It is our speculation. Therefore, we should weaken the tone of these sentences about using a higher dose. In discussion section, we have rewritten this sentence.*

*We changed “To obtain a neurological effect, we should follow a higher-dose protocol than that employed in the present study” into “We might have to follow a higher-dose level of ionized magnesium to demonstrate a favorable neurological effect.”*

Table 2: maybe specify that Postoperative ICU transfer is time 0 hr
Manuscript needs editing for clarity.

*Response: Thank you for the important point for our manuscript. We have rewritten this sentence.*

*We changed “There was no significant effect on RASS score (0 [0 to 0] vs 0 [0 to 0]; P=0.114) at postoperative ICU tranfer (Table 2). “ into “There was no significant effect on RASS score (0 [0 to 0] vs 0 [0 to 0]; P=0.114) at postoperative ICU admission, time 0 hour (Table 2). In addition, in table 2, we added “(0h)”after ICU admission.*

Reviewer #4: I would like to complement the authors with this study that, although small and negative, is important and well done. The primary outcome was whether magnesium had an effect on RASS score of patients at postoperative ICU transfer. No effect observed.

Minor comments:
Change title to:
Effect of intraoperative systemic magnesium sulphate on postoperative Richmond Agitation-Sedation Scale score after endovascular repair of aortic aneurysm UNDER GENERAL ANAESTHESIA: a doubleblind, randomized, controlled trial Short

Change short title to:
Effect of i.v. magnesium on RASS score after EVAR UNDER GENERAL ANAESTHESIA

*Response: Thank you for the important point for our manuscript. We added “under general anaesthesia” to the titles.*

The primary outcome was whether magnesium had an effect on RASS score of patients at postoperative ICU transfer. No effect observed. Likely because the study is small and therefore underpowered to detect meaningful differences

*Response: Thank you for the important point for our manuscript. And we could not show that Mg was effective for postoperative delirium and agitation. However, we planned a prospective RCT with calculating proper sample size. And we reached the conclusion that we could not obtain good results with the method of our protocol in this paper and we think that that is informative results in the field of Mg research.*

Methods: the authors state “ All P values are two-tailed.” Which is not exactly the way to formulate this. All statistical test were performed two sided.

*Response: Thank you for the important point for our manuscript. We changed “All P values are two-tailed” to “All statistical test were performed two sided” .*

Results:
“ During the observational period, no adverse events caused by magnesium infusion were
recorded, which were, for example, arrythmia, bradycardia, hypotension, effects of
muscle paralysis, convulsion and so on.” Remove the “and so on”

*Response: Thank you for the important point for our manuscript. These sentences were not detailed enough. We removed “and so on”.*

Discussion:
“ First, an administered magnesium target total of 60 mg•kg−1, actually 48±8 mg•kg−1, was not enough to obtain good outcomes” All outcomes are “ good outcomes” I think you mean that the Mg dose used might be to low tom reduce the postoperative delirium in patients undergoing EVAR surgery under general anesthesia

*Response: Thank you for the important point for our manuscript. It is our speculation. Therefore, we should weaken the tone of these sentences about using a higher dose. In discussion section, we have rewritten this sentence.*

*We changed “First, an administered magnesium target total of 60 mg•kg^−1^, actually 48±8 mg•kg^−1^, was not enough to obtain good outcomes” into “First, an administered magnesium target total of 60 mg•kg^−1^, actually 48±8 mg•kg^−1^, might not be enough to obtain good outcomes”*

Suppl figures:
Remove the operation data from your excel file… otherwise the participants are not fully anaonimsed (age surgery date and hospital could lead to a traceble study subject)

*Response: Thank you for the important point for our manuscript. Another reviewer referred to the operation management. Therefore, we remained the operation data.*

Suppl figure: Click here to access/download Supporting Information renamed_3b3df.docx Is only in jappanese state that in the suppl figure text. The Japanese supple materials can not be checked by me.

*Response: We are really sorry for your inconvenience. We showed the original version in supporting information in original language, Japanese. You can read the protocol in English version in jRCT protocol on the WEB. In addition, you can see the file “the translation of the main points of our protocol” in English in supporting information.*

7. PLOS authors have the option to publish the peer review history of their article ([what does this mean?](https://journals.plos.org/plosone/s/editorial-and-peer-review-process#loc-peer-review-history)). If published, this will include your full peer review and any attached files.

If you choose “no”, your identity will remain anonymous but your review may still be made public.

**Do you want your identity to be public for this peer review?** For information about this choice, including consent withdrawal, please see our [Privacy Policy](https://www.plos.org/privacy-policy).

Reviewer #2: No

Reviewer #3: No

Reviewer #4: **Yes:** Matijs van Meurs MD PhD

*Response: Thank you, professor Matijs van Meurs. We really appreciated that you took your time to review our paper.*
